# Ethnic differences in healthcare utilisation and diagnosis after first presentation with breathlessness: a retrospective cohort study using UK primary care records

**DOI:** 10.1038/s41533-026-00507-4

**Published:** 2026-04-03

**Authors:** Harini Sathanapally, Urvee Karsanji, Jennifer Creese, Gillian Doe, Kamlesh Khunti, Michael Steiner, Claire Lawson, Rachael A Evans, Anvesha Singh

**Affiliations:** 1 Department of Cardiovascular Sciences, University of Leicester, Leicester, UK; 2 NIHR Leicester Biomedical Research Centre, Glenfield Hospital, Leicester, UK; 3 Department of Respiratory Sciences, University of Leicester, Leicester, UK; 4 Department of Population Health Sciences, University of Leicester, Leicester, UK; 5 National Institute for Health and Care Research (NIHR) Greater Manchester Patient Safety Research Collaboration (GM PSRC), The University of Manchester, Manchester, UK; 6 Diabetes Research Centre, University of Leicester, Leicester, UK; 7 NIHR Applied Research Collaboration East Midlands, Leicester General Hospital, Leicester, UK

**Keywords:** Diseases, Health care, Medical research

## Abstract

There are known delays to diagnosis for chronic respiratory disease and recognised health inequalities in outcomes. We therefore investigated the association between ethnicity and subsequent healthcare utilisation and receipt of an explanatory diagnosis after a first presentation with breathlessness. Clinical Practice Research Datalink (CPRD) GOLD data linked to Hospital Episode Statistics (HES) and death registries were used to identify adults with a first-recorded code for breathlessness (index-date). Ethnicity was determined using the Hemingway algorithm. Rates of primary care consultations, secondary care referrals and hospital admissions within six and 24 months after index-date were examined using negative binomial regression. Logistic regression was used to estimate odds of receiving an explanatory recorded diagnosis for breathlessness during these timeframes. Models were adjusted for age, sex, socioeconomic status and ≥ 2 pre-existing long-term conditions. Amongst 88,857 included patients, 3336 were of South Asian ethnicity and 1506 of Black ethnicity. Compared to patients of White ethnicity, South Asian patients had significantly increased rates of primary care consultations and unplanned hospital admissions within six and 24 months (24 months IRR 1.13 [1.10–1.16] and 1.34 [1.25–1.45] respectively). Conversely, patients of Black ethnicity had significantly lower rates of primary care consultations within 24 months (IRR 0.95 [0.92–0.99], but significantly increased rates of unplanned hospitalisations within six and 24 months (IRR 1.33 [1.19–1.50]). However, both groups had significantly lower odds of receiving an explanatory diagnosis for breathlessness. After a first presentation with breathlessness, we observed a higher rate of unplanned hospitalisations yet a lower rate of receiving an explanatory diagnosis in patients of non-white ethnicity. Understanding the reasons and implications of these differences is critical to reduce potential health inequalities.

## Introduction

Breathlessness is a common presenting symptom in both primary and secondary care settings^1,2^. Its presence is associated with worse outcomes^3^, which increase with severity of breathlessness^4,5^, and has been shown to have prognostic value in predicting hospital admissions and mortality, independent of pre-existing cardiorespiratory comorbidities and across both hospital^4^ and community settings^5^. Whilst chronic respiratory diseases are the most common cause for chronic breathlessness, there often multiple other possible causes^6,7^. These are frequently multifactorial^8^, through an interplay of physiological, psychological, social and environmental factors^9^. The assessment and clinical decision-making processes relating to breathlessness can therefore be complex^10^, with a known association with increased health-service utilisation after presentation^4,5,11^, and diagnostic delays^6,10,12^ and misdiagnoses^12,13^ being common.

Equitable healthcare pathways to ensure accurate diagnoses and support for patients with breathlessness are two of the top 10 priorities identified by patients, carers and clinicians through the UK James Lind Alliance priority setting partnership^14^. Ethnicity is a known driver of health inequalities, which have been defined as preventable differences in health, and in the experience of healthcare, between different groups of people^15^. People from ethnic minority backgrounds have previously been identified as being at increased risk of experiencing inequalities in access to healthcare^16,17^, and the quality of care received^18,19^. Differences in the reported experience of breathlessness among different sociodemographic groups have previously been described, and people of non-White ethnicity are more likely to report experiencing breathlessness^20–24^, and to experience poorer asthma outcomes compared with patients of White ethnicity^25,26^. However, the influence of ethnicity on the healthcare pathway after presentation with breathlessness has not been investigated and is a crucial step in identifying potential areas of disparity and health inequity.

Therefore, the aim of this study was to investigate the influence of ethnicity on the healthcare pathway after a coded first presentation with breathlessness in primary care records, including incidence rates of primary care consultations, outpatient referrals into secondary care, hospital admissions, and receipt of coded explanatory diagnosis.

## Methods

### Study design and participants

This was a retrospective cohort study using data from the Clinical Practice Research Datalink (CPRD) GOLD database, which is a national database containing anonymised data including Read codes entered as part of clinical documentation in consenting GP practices across England^27^. Data from CPRD GOLD were linked to Hospital Episode Statistics (HES) Admitted Patient Care (APC) database for hospital admissions, the 2015 English Index of Multiple Deprivation (IMD) database for socioeconomic status at a residential area level, and Office for National Statistics (ONS) for mortality data.

**Fig. 1:**
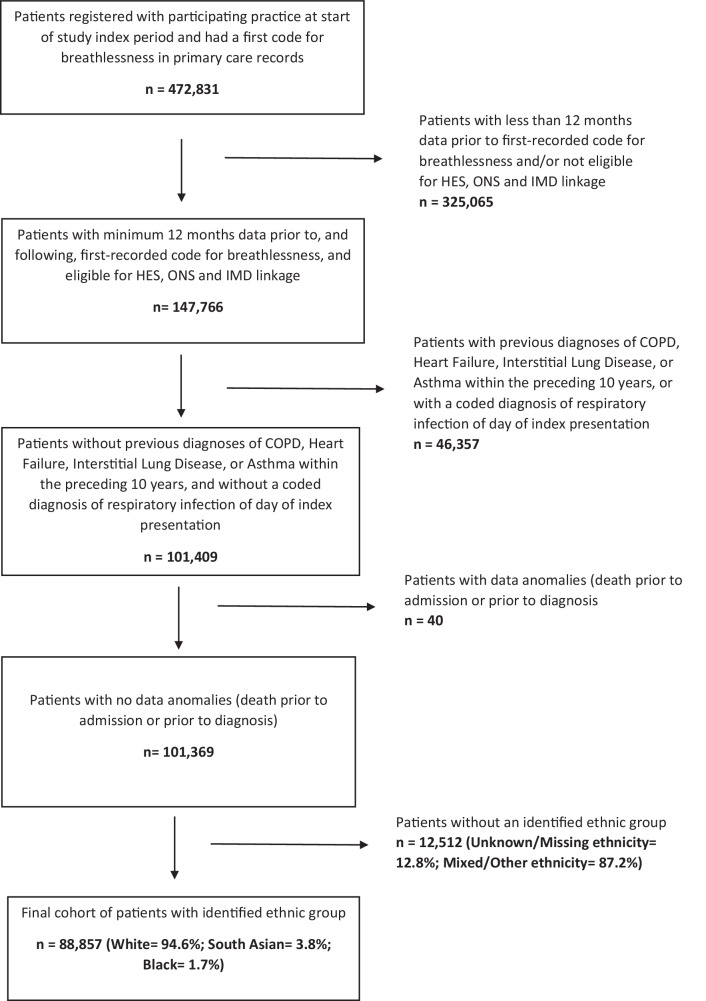
Study patient selection flowchart - each panel shows the number of patients at each stage of the study, side arrows indicate the number of patients excluded at each stage, along with reasons for exclusion.

We identified codes for breathlessness in CPRD using a list of 36 read codes previously published by Watson et al. ^28^ (Section 1, Supplementary file 1). Adults (aged 18 or over) with a first-recorded code for breathlessness between 1^st^ January 2007 and 31^st^ December 2017, and without any previously recorded codes for breathlessness prior to 1^st^ January 2007, were identified and included^29^. There is no specific code for chronic breathlessness, therefore we made a series of criteria to enrich for *first* presentations of chronic breathlessness. Firstly, patients with any previously coded diagnoses for chronic cardiorespiratory diseases which commonly cause breathlessness, were excluded. These included interstitial lung disease (ILD), heart failure (HF), chronic obstructive pulmonary disease (COPD). Asthma was excluded if coded in the preceding 10 years only, to avoid exclusion of patients with an inactive unrelated diagnosis of asthma (for example in childhood). In previous work using the same cohort, a sensitivity analysis including any pre-existing potential explanatory diagnosis did not affect the results^29^ Secondly, patients who had a coded diagnosis of acute respiratory infection on the same date as their coded presentation with breathlessness were also excluded, as a surrogate strategy to exclude presentations with breathlessness which were likely to be acute or transient (Data flowchart Fig. 1, Code list Section 4, Supplementary file 1). This is likely to be more common than the small number of acute on chronic presentations of breathlessness that would also be excluded. Thirdly, we performed sensitivity analyses on a cohort of patients with two or more episodes coded for breathlessness.

### Outcomes

Outcomes included all-cause rates of primary care consultations, routine and urgent referrals into secondary care, and unplanned hospital admissions, as well as receipt of a coded potential explanatory diagnosis for breathlessness. Data pertaining to consultations and outpatient referrals were extracted using the ascertainment code set (Section 5, Supplementary file 1) from all available records. Hospital admissions data for all causes were extracted through HES APC. Primary care consultations for all causes conducted in a general practice setting (including clinic, telephone and home visit) were identified and recorded as counts. Repeat consultation entries on the same day were excluded to avoid duplication. Clinician referrals into outpatient secondary care for all causes were identified and grouped into those coded as urgent, and those coded as routine in CPRD. A list of potentially explanatory diagnoses (including respiratory, cardiac, mental health, obesity and neuromuscular diagnoses) was made from existing literature reported elsewhere^7,29^. Diagnoses could have been made in primary care or secondary care and were ascertained from CPRD data (which includes diagnoses recorded in primary care and also those communicated to primary care from secondary care) as well as linked HES APC data, which captures diagnoses recorded during hospital admissions. Diagnoses were identified through Read codes in CPRD and International Classification of Diseases-10 (ICD10) codes in HES APC (Code list Section 2 & 3, Supplementary file 1).

**Fig. 2:**
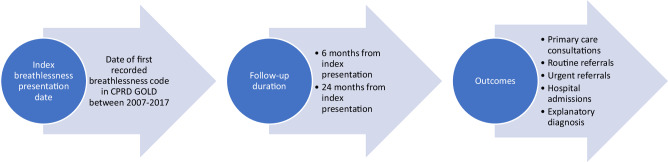
Study timeline and outcomes.

Follow-up was over two timeframes: six months (to reflect the target timeframe recommended by NHS England for diagnosis and management plan formulation for people presenting with breathlessness^6^), and 24 months (to allow a longer follow-up period to establish a diagnosis for breathlessness and allow for delays in diagnostic pathways), from the index presentation with breathlessness (Fig. 2).

### Variables

The key variable of interest was ethnicity, which was determined through CPRD and HES records using the Hemingway algorithm^30^. In this approach, the ethnicity as coded in CPRD is prioritised, and in the event of there being more than one recorded ethnicity code in CPRD, the most frequent code is used, or the more recent code if the two most frequent codes appear equally. To facilitate interpretability in the primary analyses, we chose a priori to only include patients who could be assigned to an ethnic group after application of the Hemingway algorithm. However, we conducted further sensitivity analyses including patients with ethnicity coding of “Mixed/other” or “Unknown/Missing”.

Covariates included age, sex, IMD and the presence of underlying MLTC (to mitigate the presence of other underlying conditions that may have led to a predisposition for increased health service utilisation and diagnosis amongst our patient cohort). Age was included as a continuous variable and sex was categorised as “Male” and “Female”. Socio-economic status was defined using the Index of Multiple Deprivation (IMD), which is a patient-level score derived through a weighted aggregation of multiple indicators of deprivation based on residential area, including crime, employment and living conditions^31^. The IMD score was categorised into quintiles with IMD 1 representing the area of lowest deprivation and IMD 5 indicating the area of highest. In line with the widely accepted definition of multimorbidity, now referred to as multiple long-term conditions (MLTC)^32,33^, we defined the presence of underlying MLTC as coded pre-existing diagnoses of two or more long-term conditions at the point of index presentation with breathlessness. Coded diagnoses of long-term conditions were extracted using Read codes translated to medcodes in CPRD, in accordance with those listed in the Cambridge Multimorbidity Score (CMS)^34^ (included conditions listed in Section 6, Supplementary file 1).

### Statistical analysis

Descriptive statistics were used to summarise patient characteristics and frequency counts of outcome events. Negative binomial regression was used to model rates of primary care consultations, referrals into secondary care and hospital admissions, and estimate incidence rate ratios (IRR) with 95% confidence intervals (CI), comparing patients of South Asian and Black ethnicities to patients of White ethnicity as the reference ethnic group.

Unadjusted IRR were modelled before adjustment with age, sex and IMD and then further adjustment for presence of pre-existing coded diagnoses of underlying MLTC. Logistic regression was used to estimate unadjusted and adjusted odds ratios (OR) with 95% CI for receiving an explanatory diagnosis for breathlessness following the index date and comparing the same groups.

We conducted further sensitivity analyses to test the robustness of our findings. We repeated the final analyses (Sensitivity analysis 1) in a cohort of patients who had at least one further recorded code for breathlessness after their index presentation (to further enrich the cohort for “chronic breathlessness”). We also repeated analyses with inclusion of patients with Mixed/Other and Unknown/Missing ethnicities (Sensitivity analysis 2) to assess whether exclusion of these groups in the primary analyses may have affected the direction and significance of the results.

### Ethical approval

This study was approved by the Independent Scientific Advisory Committee for Medicines and Healthcare products Regulatory Agency database research (Protocol Number = 20_075). This study is based in part on data from the Clinical Practice Research Datalink obtained under licence from the UK Medicines and Healthcare products Regulatory Agency. Data use complies with the UK Data Protection Act 2018, the General Data Protection Regulation (GDPR), and the ethical principles of the Declaration of Helsinki.

## Results

### Baseline characteristics

We identified 101,369 patients with a first recorded code for breathlessness between 2007-2017, of whom 88,857 had an identifiable ethnic group through the Hemingway algorithm and were included. Patients without an identifiable ethnic group i.e., Mixed/Other (*n* = 1603) or Unknown/Missing (*n* = 1389) were excluded from primary analyses.

Of the 88,857 patients who met the inclusion criteria, 94.6% (*n* = 84015) were of White ethnicity, 3.8% (*n* = 3336) of South Asian ethnicity and 1.7% (*n* = 1506) of Black ethnicity. Patients of South Asian and Black ethnicities were significantly younger than patients of White ethnicity (median age 47, 48, and 61 years respectively) (Table 1). A higher proportion of those from ethnic minority groups were from the most deprived areas (IMD 5): 24% South Asian and 39% Black, compared to 16% White (Table 1).

**Table 1:** Baseline characteristics, data are presented as median (IQR) for continuous measures, and *n* (%) for categorical measures.

Ethnicity		White	South Asian	Black	Mixed/Other	Unknown/Missing	p value
Total (n)		84,015	3336	1506	1603	10,909	
Median age (IQR)		61.0 (47.0-72.0)	47.0 (36.0-61.0)	48.0 (39.0-60.0)	49.00 (38.00-62.00)	57.0 (45.0-67.0)	<0.001^b^
IMD n (%)	1 (lowest deprivation)	18742 (22.3%)	593 (17.8%)	108 (7.2%)	320 (20.0%)	3202 (29.4%)	<0.001^a^
	2	18499 (22.0%)	624 (18.7%)	135 (9.0%)	300 (18.7%)	2648 (24.3%)	
	3	17661 (21.0%)	644 (19.3%)	274 (18.2%)	322 (20.1%)	2205 (20.2%)	
	4	15293 (18.2%)	678 (20.3%)	405 (26.9%)	304 (19.0%)	1605 (14.7%)	
	5 (highest deprivation)	13781 (16.4%)	797 (23.9%)	584 (38.8%)	356 (22.2%)	1242 (11.4%)	
MLTC n (%)	Absent	34332 (40.9%)	1868 (56.0%)	858 (57.0%)	886 (55.3%)	6262 (57.4%)	<0.001^a^
	Present	49683 (59.1%)	1468 (44.0%)	648 (43.0%)	717 (44.7%)	4647 (42.6%)	
Sex n (%)	Male	37596 (44.8%)	1411 (42.3%)	553 (36.7%)	702 (43.8%)	5050 (46.3%)	<0.001^a^
	Female	46419 (55.3%)	1925 (57.7%)	953 (63.3%)	901 (56.2%)	5859 (53.7%)	
Smoking status n (%)	Non- smoker	31683 (37.7%)	2482 (74.4%)	951 (63.2%)	849 (53.0%)	4889 (44.8%)	<0.001^a^
	Ex- smoker	32204 (38.3%)	440 (13.2%)	304 (20.2%)	414 (25.8%)	3812 (34.9%)	
	Current smoker	18021 (21.5%)	359 (10.8%)	227 (15.1%)	306 (19.1%)	1959 (18.0%)	
	Missing	2107 (2.5%)	55 (1.7%)	24 (1.6%)	34 (2.1%)	249 (2.3%)	

*IMD* index of multiple deprivation, *MLTC* multiple long-term conditions.

^a^Chi-square test.

^b^Kruskal-Wallis test.

### Primary care consultations after index presentation with breathlessness

**Fig. 3:**
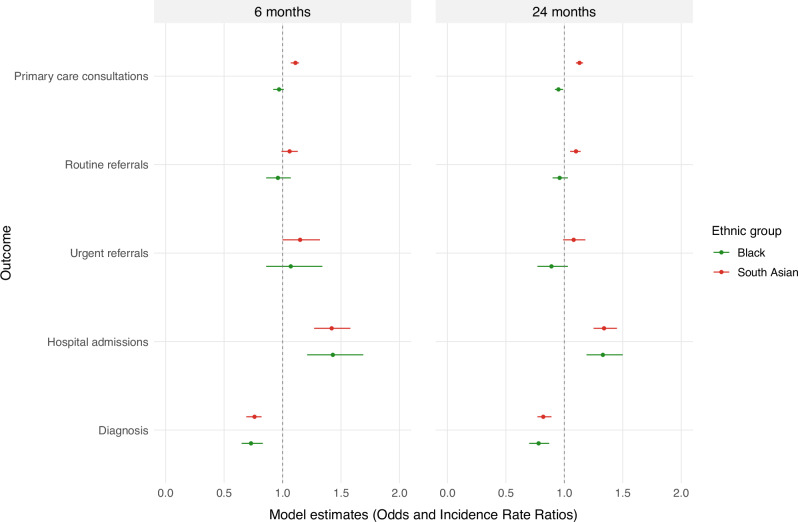
Associations between ethnic group (Black and South Asian, compared to White) and odds of diagnosis and incidence rates of healthcare utilisation. All estimates adjusted for age, sex, index of multiple deprivation and presence of underlying multiple long-term conditions. Dotted line shows a ratio of 1.0 indicating no association.

Adjusted models showed a significantly increased IRR for primary care consultations within six months from index presentation in patients of South Asian ethnicity (IRR 1.11 95% CI 1.07 to 1.14) (Table 3, Fig. 3), compared with patients of White ethnicity. Whilst a significantly decreased IRR for primary care consultations within six months from index presentation was observed for patients of Black ethnicity in models adjusted for age, sex and deprivation(IRR 0.94 95% CI 0.90 to 0.98) (Fig. 3, Table S1 Supplementary file 2), further adjustment for presence of MLTC showed no statistically significant difference in IRR 0.97 (0.92 to 1.01) (Table 3, Fig. 3).

Within 24 months from index presentation, adjusted models showed a significantly increased IRR for primary care consultations in patients of South Asian ethnicity (IRR 1.13 95% CI 1.10 to 1.16), but a significantly decreased IRR in patients of Black ethnicity (IRR 0.95 95% CI 0.92 to 0.99) (Table 3, Fig. 3), compared to patients of White ethnicity. Sensitivity analyses 1 & 2 resulted in maintenance of the direction and statistical significance of associations in both patient groups, within both follow-up timeframes (Sensitivity analysis 1 & 2, Table S1, Supplementary file 2).

### Routine outpatient secondary care referrals after index presentation with breathlessness

Adjusted models did not show any significant differences in IRR for routine referrals sent for patients of Black (IRR 0.96 95% CI 0.86 to 1.07) or South Asian (IRR 1.06 95% CI 0.99 to 1.13) ethnicities compared with patients of White ethnicity, within six months from index presentation (Fig. 3; Table 3).

However, significantly increased IRR of routine referrals were observed within 24 months from index date in patients of South Asian ethnicity (IRR 1.10 95% CI 1.05 to 1.14), whilst no significant difference was observed in patients of Black ethnicity in adjusted models (IRR 0.96 95% CI 0.90 to 1.03) (Fig. 3; Table 3). Sensitivity analysis 1 resulted in the IRR within six months for patients of South Asian ethnicity reaching statistical significance, with the direction of the estimate maintained. There were no other changes in direction or significance of the results from the primary analyses in both Sensitivity analysis 1 &2 (Sensitivity analysis 1, Table S2, Supplementary file 2).

### Urgent outpatient secondary care referrals after index presentation with breathlessness

Within six-months from index presentation, adjusted models did not show any significant differences in IRR of urgent referrals generated for patients of Black (IRR 1.07 95% CI 0.86 to 1.34) or of South Asian (1.15 95% CI 1.00 to 1.32) ethnicities, compared with patients of White ethnicity (Table 3, Fig. 3).

Within 24-months from index presentation, adjusted models again did not show any significant differences in IRR of urgent referrals generated for patients of South Asian (IRR 1.08 95% CI 0.99 to 1.18) or Black (IRR 0.89 95% CI 0.77 to 1.03) ethnicities (Table 3, Fig. 3). Sensitivity analysis 1 resulted in the IRR reaching statistical significance within both timeframes for patients of South Asian ethnicity, with the direction of the estimate maintained. There were no other changes in direction or significance of the results from the primary analyses in both Sensitivity analysis 1 &2 (Sensitivity analysis 1, Table S3, Supplementary file 2).

### Unplanned hospital admissions after index presentation with breathlessness

Within six months from index presentation, adjusted models showed significantly increased IRR for unplanned hospital admissions for patients of South Asian ethnicity (IRR 1.42 95% CI 1.27 to 1.58) and patients of Black ethnicity (IRR 1.43 95% CI 1.21 to 1.69) (Fig. 3; Table 3).

At 24 months from index presentation, adjusted models again showed a significantly increased IRR for unplanned hospital admissions for patients of South Asian ethnicity (IRR 1.34 95% CI 1.25 to 1.45) and for patients of Black ethnicity (IRR 1.33 95% CI 1.19 to 1.49) (Fig. 3; Table 3).

Sensitivity analyses resulted in a maintenance of the direction and statistical significance of associations in both patient groups, within both follow-up timeframes (Sensitivity analysis 1, Table S4, Supplementary file 2).

### Receipt of an explanatory diagnosis after index presentation with presentation with breathlessness

Compared with patients of White ethnicity, patients of South Asian ethnicity and Black ethnicity were significantly less likely to receive an explanatory diagnosis within six months in all models (OR 0.76 95% CI 0.69 to 0.82), (OR 0.73 0.65 to 0.83), respectively (Fig. 3, Table 3). Compared with patients of White ethnicity, patients of South Asian ethnicity and Black ethnicity remained significantly less likely to receive an explanatory diagnosis within 24 months from index presentation in all models (OR 0.82 95% CI 0.77 to 0.89), (OR 0.78 95% CI 0.70 to 0.87), respectively (Fig. 3; Table 3).

Sensitivity analyses showed no change in the direction nor the statistical significance of associations in both patient groups within both follow-up timeframes (Sensitivity analysis 3, Table S5, Supplementary file 2).

## Discussion

We report important differences between ethnic groups in healthcare utilisation, clinician referrals into secondary care and likelihood of receiving an explanatory diagnosis within six months and two years after a first presentation with breathlessness. Patients of South Asian and Black ethnicities were significantly younger and significantly less likely to have MLTC, compared to patients of White ethnicity (Table 2). Nevertheless, patients of South Asian ethnicity had significantly higher rates of primary care consultations within both six months and 24 months, and of routine referral into secondary care within 24 months after presentation with breathlessness, compared to patients of White ethnicity. Conversely, patients of Black ethnicity had significantly lower rates of primary care consultations within 24 months from presentation with breathlessness. Despite these differences, both groups had significantly higher rates of unplanned hospital admissions yet lower odds of receiving an explanatory diagnosis for breathlessness within six and 24 months, compared with patients of White ethnicity (Table 3).

**Table 2:** Distributions of patients by outcome, including proportions within each ethnic group.

Outcome	Follow up duration	Frequency	Total (*n* = 88,857)	White (*n* = 84,015)	South Asian (*n* = 3336)	Black (*n* = 1506)
Primary care consultations (n%)	6 months	1 or more	83525 (94.0%)	79020 (94.1%)	3120 (93.5%)	1385 (92.0%)
		3 or more	67996 (76.5%)	64381 (76.6%)	2525 (75.7%)	1090 (72.4%)
	24 months	1 or more	88375 (99.5%)	83569 (99.5%)	3307 (99.1%)	1499 (99.5%)
		3 or more	85750 (96.5%)	81082 (96.5%)	3212 (96.3%)	1456 (96.7%)
Routine referrals (n%)	6 months	1 or more	45111 (50.8%)	42538 (50.6%)	1779 (53.3%)	794 (52.7%)
		3 or more	21021 (23.7%)	19641 (23.4%)	929 (27.8%)	451 (29.9%)
	24 months	1 or more	67790 (76.3%)	63990 (76.2%)	2632 (78.9%)	1168 (77.6%)
		3 or more	30805 (34.7%)	28960 (34.5)	1276 (38.2%)	569 (37.8%)
Urgent referrals (n%)	6 months	1 or more	25425 (28.6%)	23826 (28.4%)	1081 (32.4%)	518 (34.4%)
		3 or more	19431 (21.9%)	18124 (21.6%)	873 (26.2%)	434 (28.8%)
	24 months	1 or more	33435 (37.6%)	31503 (37.5%)	1334 (40.0%)	598 (39.7%)
		3 or more	19857 (22.3%)	18530 (22.1%)	887 (26.6%)	440 (29.2%)
Hospital admissions (n%)	6 months	1 or more	46980 (52.9%)	44043 (52.4%)	1988 (59.6%)	949 (63.0%)
		3 or more	38791 (43.7%)	36327 (43.2%)	1667 (50.0%)	797 (52.9%)
	24 months	1 or more	57979 (65.2%)	54429 (64.8%)	2429 (72.8%)	1121 (74.4%)
		3 or more	41281 (46.5%)	38631 (46.0%)	1787 (53.6%)	863 (57.3%)
Diagnosis received (n%)	6 months	1 or more	25775 (29%)	24737 (29.4%)	711 (21.3%)	327 (21.7%)
	24 months	1 or more	39302 (44.2%)	37553 (44.6%)	1204 (36.09%)	545 (36.2%)

**Table 3:** Adjusted results from negative binomial regression models (incidence ratio ratio) and logistic regression models (odds ratio), all models adjusted for age, sex, ethnic group, deprivation score (IMD) and presence of MLTC, * *p* < 0.05, ** *p* < 0.01, *** *p* < 0.001.

Variable		Primary care consultations (incidence rate ratio 95% CI)		Routine referrals (incidence rate ratio 95% CI)		Urgent referrals (incidence rate ratio 95% CI)		Hospital admissions (incidence rate ratio 95% CI)		Likelihood of diagnosis (Odds ratio 95% CI)	
		6 months	24 months	6 months	24 months	6 months	24 months	6 months	24 months	6 months	24 months
Age		1.01 (1.01 to 1.01)***	1.01 (1.01 to 1.01)***	1.01 (1.01 to 1.01)***	1.00 (1.00 to 1.01)***	1.02 (1.01 to 1.02)***	1.01 (1.01 to 1.01)***	0.99 (0.99 to 0.99)***	0.99 (0.99 to 1.00)***	1.02 (1.02 to 1.02)***	1.02 (1.01 to 1.02)***
Sex	Male	Ref 1.0	Ref 1.0	Ref 1.0	Ref 1.0	Ref 1.0	Ref 1.0	Ref 1.0	Ref 1.0	Ref 1.0	Ref 1.0
	Female	1.10 (1.09 to 1.11)***	1.15 (1.14 to 1.16)***	0.98 (0.96 to 1.00)	1.05 (1.39 to 1.07)***	0.90 (0.88 to 0.95)***	1.02 (0.99 to 1.05)	1.04 (1.00 to 1.10)	1.01 (0.98 to 1.04)	0.81 (0.79 to 0.84)***	0.90 (0.87 to 0.92)***
IMD	1 (least deprived)	Ref 1.0	Ref 1.0	Ref 1.0	Ref 1.0	Ref 1.0	Ref 1.0	Ref 1.0	Ref 1.0	Ref 1.0	Ref 1.0
	2	0.99 (0.98 to 1.01)	1.00 (0.98 to 1.01)	0.97 (0.94 to 1.01)	0.98 (0.96 to 1.00)	0.95 (0.88 to 1.03)	0.97 (0.93 to 1.02)	0.98 (0.91 to 1.05)	0.99 (0.94 to 1.03)	1.07 (1.02 to 1.11)**	1.08 (1.03 to 1.12)***
	3	1.02 (1.00 to 1.04)	1.02 (1.00 to 1.04)*	0.93 (0.89 to 0.96)***	0.94 (0.91 to 0.96)***	0.97 (0.90 to 1.05)	0.96 (0.91 to 1.01)	0.97 (0.91 to 1.04)	1.05 (1.00 to 1.10)*	1.11 (1.06 to 1.16)***	1.12 (1.08 to 1.17)***
	4	1.01 (1.00 to 1.03)	1.01 (0.99 to 1.03)*	0.92 (0.89 to 0.95)***	0.93 (0.90 to 0.95)***	0.89 (0.82 to 0.96)**	0.91 (0.86 to 0.96)***	0.91 (0.84 to 0.97)**	1.03 (0.98 to 1.07)	1.21 (1.15 to 1.27)***	1.26 (1.20 to 1.31)***
	5 (most deprived)	0.99 (0.98 to 1.01)	1.02 (1.00 to 1.03)*	0.96 (0.92 to 0.99)*	0.97 (0.94 to 0.99)**	0.91 (0.83 to 0.98)*	0.93 (0.88 to 0.98)**	0.95 (0.89 to 1.02)	1.09 (1.04 to 1.14)**	1.40 (1.33 to 1.47)***	1.42 (1.36 to 1.49)***
MLTC	Absent	Ref 1.0	Ref 1.0	Ref 1.0	Ref 1.0	Ref 1.0	Ref 1.0	Ref 1.0	Ref 1.0	Ref 1.0	Ref 1.0
	Present	1.37 (1.35 to 1.38)***	1.48 (1.47 to 1.50)***	1.14 (1.12 to 1.17)***	1.25 (1.23 to 1.27)***	1.16 (1.10 to 1.22)***	1.34 (1.29 to 1.39)***	1.14 (1.08 to 1.20)***	1.31 (1.27 to 1.35)***	1.15 (1.12 to 1.19)***	1.44 (1.39 to 1.48)***
Ethnic group	White	Ref 1.0	Ref 1.0	Ref 1.0	Ref 1.0	Ref 1.0	Ref 1.0	Ref 1.0	Ref 1.0	Ref 1.0	Ref 1.0
	South Asian	1.11 (1.07 to 1.14)***	1.13 (1.10 to 1.16)***	1.06 (0.99 to 1.13)	1.10 (1.05 to 1.14)***	1.15 (1.00 to 1.32)	1.08 (0.99 to 1.18)	1.42 (1.27 to 1.58)***	1.34 (1.25 to 1.45)***	0.76 (0.69 to 0.82)***	0.82 (0.77 to 0.89)***
	Black	0.97 (0.92 to 1.01)	0.95 (0.92 to 0.99)*	0.96 (0.86 to 1.07)	0.96 (0.90 to 1.03)	1.07 (0.86 to 1.34)	0.89 (0.77 to 1.03)	1.43 (1.21 to 1.69)***	1.33 (1.19 to 1.50)***	0.73 (0.65 to 0.83)***	0.78 (0.70 to 0.87)***

*IRR* incidence rate ratio, *IMD* index of multiple deprivation, *MLTC* multiple long-term conditions, *OR* odds ratio, *CI* confidence interval.

### Comparison with previous literature

In comparison to the overall CPRD GOLD population with a recorded ethnicity code, we observed a higher proportion of patients of White ethnicity and a lower proportion of patients of South Asian and Black ethnicities in our cohort of patients with an index first presentation with breathlessness^35^. Previous work has shown that CPRD-HES linked data are generally representative of the UK population, with high completeness and strong agreement with HES^35,36^. The ethnicity distribution in our cohort therefore appears to differ, and is in contrast to previous literature reporting that people of non-white ethnicity are more likely to report experiencing breathlessness^20–24^. This apparent difference may reflect selection factors related to healthcare presentation or differences in recording practices.

Ethnicity based differences in rates of referral from primary care into secondary care have been previously reported in the context of mental health^37^, where adult patients of Black African and African Caribbean ethnicity^38^, and of Black British and Asian ethnicity^39^ have been found to be less likely to be referred to mental health services via GP practices, compared to patients of White ethnicity. Lower rates of referral into pulmonary rehabilitation for patients of Black ethnicity have also been previously described in the context of COPD^40^. However, our findings showed no significant differences in IRR of referral into secondary care for all causes in patients of Black ethnicity, and in contrast noted significantly higher IRR of routine referrals for all causes in patients of South Asian ethnicity.

Our findings suggest that patients of South Asian and Black ethnicity are less likely to receive any explanatory diagnosis for breathlessness despite the overall higher rate of accessing healthcare. Ethnic minority groups have previously been described to be more likely to experience delayed or missed diagnoses in the contexts of dementia, breast cancer, cardiovascular and rheumatological disease^41–45^. Whilst it is difficult to draw further conclusions about the reasons for these findings from our dataset, potential explanations include differences in how breathlessness is communicated to or interpreted by healthcare professionals^46^, lower rates of investigations, or underlying causes for breathlessness that may not be routinely coded.

Our findings are in line with previous reports from a study of ethnicity-based differences in early onset MLTCs using CPRD GOLD data, in which patients of South Asian ethnicity had the highest median number of primary care consultations per individual, and patients of Black ethnicity had the lowest^47^. One previous study in the context of Asthma reported that patients of Black ethnicity had higher rates of hospitalisation due to asthma exacerbations^48^, and another study in the context of COPD found patients of Black ethnicity to have an increased risk of hospitalisation with respiratory causes^40^. Another study found patients of Asian and Black ethnicities to have an increased risk of all-cause hospital admissions after presentation with any acute illness^49^. We also noted an increased risk of all cause hospital admissions for patients of South Asian and Black ethnicities within six and 24 months after presentation with breathlessness, and found that this risk was highest within the six-month timeframe after presentation with breathlessness. This is of concern as unplanned hospital admissions frequently reflect major upheaval, with adverse consequences for patients and for the health service^50^.

These results along with our finding of variable rates of primary care consultation patterns in these patient groups, suggest there may be specific cultural factors influencing patterns of health service utilisation. Indeed, a previous study of 1098 English general practices found significantly worse ratings of primary care services by patients of non-White ethnicity^51^. Differences persisted after adjustment for waiting times suggesting a potential difference in expectations. There was some mitigation for patients of Black and Chinese ethnicities after adjustment for staff communication skills, highlighting the role of potential communication issues^51^. Further in-depth exploration of these factors in relation to presentations with breathlessness are needed to better understand the underlying mechanisms and implications of our data findings.

### Strengths and limitations

A key strength of this study is the use of a large representative database with real-world longitudinal data spanning primary and secondary care. This enabled longitudinal examination in clinical practice and the observation of real-life trends along the healthcare pathway after presentation with breathlessness across different ethnic groups. Through the use of robust statistical analyses with adjustment for potential confounding variables, we have been able to highlight significant differences in healthcare utilisation and likelihood of receiving an explanatory diagnosis, which may be contributing to potential health inequity and warrant further exploration. Without a diagnosis patients will not be receiving treatment for the underlying disease, exercise rehabilitation or symptom management. Further sensitivity analyses, including analyses in a cohort of patients who had at least one further recorded code for breathlessness after their index presentation (to further enrich the cohort for “chronic breathlessness”) reinforced the direction and significance of our findings.

However, there are also noteworthy limitations. First, there is no routinely used agreed code for chronic breathlessness in primary care nor secondary care (at the time of this dataset) and symptom duration prior to presentation is also currently not captured through clinical coding. We therefore needed to make some assumptions around coding. Secondly, there are likely to be patients presenting with breathlessness who are not captured in our cohort due to heterogeneity in coding practices. CPRD data is obtained through extraction of Read codes from practices mostly using the ViSion (ViSion INPS, London, UK) software for clinical documentation, which requires clinicians to enter a Read code prior to entering the consultation notes. However, the notes themselves beyond this point can be entered as free text, which may mean that some symptom presentations are captured in the free text rather than in the form of a Read code, such as where the symptom was not the primary reason for presentation, or where clinicians judge the symptom to be “lower risk” compared to others the patient may be experiencing^52^. As it was not possible for us to access to the free text of consultation notes through CPRD for this study, it was not possible to capture presentations of breathlessness recorded in free text form only, and the impact of this omission on the outcomes of this study are as yet unknown.

Third, through adjustment for the presence of MLTC in our final models, we attempted to mitigate the presence of other underlying conditions that may have led to a predisposition for increased health service utilisation amongst our patient cohort, however there may be other unmeasured confounding. Fourth, given the multifactorial aetiology of breathlessness and the issues with symptom coding, we chose a priori to use all-cause primary care consultations, referrals and hospital admissions. Fifth, patients with ethnicity coded as Mixed/Other (1.6%), or Unknown/Missing (10.7%) were excluded from our primary analyses. Ethnicity recording in CPRD is less detailed than in ONS Census data, where ethnic subgroups are separately enumerated. As a result, we were unable to examine outcomes in Mixed/Other groups, nor explore finer-grained categories within White, South Asian, and Black ethnicities. However, in sensitivity analyses where patients with Mixed/Other and Unknown/Missing ethnicities were included as separate categories, results were consistent with our main findings, suggesting that this exclusion is unlikely to have materially altered conclusions. Finally, we were unable to capture severity of breathlessness in this study, as measures of breathlessness severity are usually documented during chronic disease reviews rather than at the point of first presentation, and therefore could not be reliably analysed for the purposes of this study.

## Conclusion

Our findings have highlighted significant differences in patterns of health care utilisation and outcomes after presentation with breathlessness coded in primary care records, in people of South Asian and Black ethnicity compared to patients of White ethnicity. Compared with patients of white ethnicity, patients of South Asian ethnicity had a higher rate of primary care consultations whereas patients of Black ethnicity had a lower rate. Despite these differences, both groups had a higher rate of future unplanned hospital admissions and a lower likelihood of receiving an explanatory diagnosis for breathlessness compared to people of White ethnicity. Our findings demonstrate the need for further research to understand the underlying factors that may be contributing to these differences, including how breathlessness is perceived, relevant health behaviours, healthcare professional views and behaviours, and experiences of healthcare received after presentation with breathlessness. The implications on future outcomes need urgent investigation due to potential, currently hidden, health inequalities.

## Supplementary information

Supplementary file 1

Supplementary file 2

## Data Availability

The protocol for this study was approved by the Independent Scientific Advisory Committee (ISAC) for MHRA Database Research (protocol number 20_075). This study uses data from CPRD obtained under licence from the UK Medicines and Healthcare products Regulatory Agency (MHRA). CPRD data are not publicly available and access requires approval from CPRD. The data are provided by patients and collected by the National Health Service (NHS) as part of their care and support. Informed consent from individual patients is not required, as CPRD manages anonymised, routinely collected health data. Data use complies with the UK Data Protection Act 2018, the General Data Protection Regulation (GDPR), and the ethical principles of the Declaration of Helsinki. Linked pseudonymised mortality data from the Office for National Statistics (ONS), socioeconomic data from the Index of Multiple Deprivation (IMD), and secondary care data from Hospital Episode Statistics (HES) were provided for this study by CPRD for patients in England. Data linkage is performed by NHS Digital, the statutory trusted third party, using identifiable data held only by NHS Digital. General practices consent to this process at the practice level, and individual patients have the right to opt out.
