# Supplementary material for: Ethnic differences in healthcare utilisation and diagnosis after first presentation with breathlessness: a retrospective cohort study using UK primary care records
Source: NPJ Prim Care Respir Med. 2026 Apr 3;36:50. doi: 10.1038/s41533-026-00507-4 (PMC13421450; doi:10.1038/s41533-026-00507-4)
Supplement: Supplementary file 2 [file 41533_2026_507_MOESM2_ESM.docx]

Contents

[Table S1 Negative binomial regression to compare incidence rate ratios (IRR 95% CI ) for rates of primary care consultations across groups 1](#_Toc207721593)

[Table S2 Negative binomial regression to compare incidence rate ratios (IRR 95%CI) for rates of routine referrals into secondary care across groups 2](#_Toc207721594)

[Table S3 Negative binomial regression to compare incidence rate ratios (IRR 95%CI) for rates of urgent referrals across groups 3](#_Toc207721595)

[Table S4 Negative binomial regression to compare Incidence rate ratios (IRR 95%CI) for rates of hospital admissions across groups 4](#_Toc207721596)

[Table S5 Logistic regression to compare likelihood of diagnosis (OR 95%CI) across groups 5](#_Toc207721597)

|  | **Unadjusted IRR (95%CI)** | | **Adjusted IRR (age, sex, ethnicity, IMD)** | | **Adjusted IRR (95%CI) (age, sex, ethnicity, IMD, MLTC)** | | **Sensitivity analysis 1 Adjusted IRR (95%CI) (age, sex, ethnicity, IMD, MLTC)** | | **Sensitivity analysis 2 Adjusted IRR (95%CI) (age, sex, ethnicity, IMD, MLTC)** | |
| --- | --- | --- | --- | --- | --- | --- | --- | --- | --- | --- |
| **Variable** | **Within 6 months** | **Within 24 months** | **Within 6 months** | **Within 24 months** | **Within 6 months** | **Within 24 months** | **Within 6 months** | **Within 24 months** | **Within 6 months** | **Within 24 months** |
| **Age** | 1.01 (1.01 to 1.01) *** | 1.01 (1.014 to 1.014) | 1.01 (1.01 to 1.01)*** | 1.01 (1.01 to 1.01)*** | 1.01 (1.01 to 1.01)*** | 1.01 ( 1.01 to 1.01)*** | 1.01 (1.01 to 1.01)*** | 1.01 (1.01 to 1.01)*** | 1.01 (1.01,1.01)*** | \| 1.01 \| \| --- \| \| (1.01,1.01)*** \| |
| **Male** | Ref 1.0 | Ref 1.0 | Ref 1.0 | Ref 1.0 | Ref | Ref 1.0 | Ref 1.0 | Ref 1.0 | Ref 1.0 | Ref 1.0 |
| **Female** | 1.08 (1.07 to 1.09)*** | 1.12 (1.11 to 1.13) | 1.11 (1.10 to 1.13)*** | 1.17 (1.16 to 1.18)*** | 1.10 (1.09 to 1.11)*** | 1.15 ( 1.14 to 1.16)*** | 1.07 (1.05 to 1.09)*** | 1.11 (1.09 to 1.12)*** | 1.10***(1.09,1.11) | 1.15***(1.14,1.16) |
| **IMD 1 (lowest deprivation)** | Ref 1.0 | Ref 1.0 | Ref 1.0 | Ref 1.0 | Ref 1.0 | Ref 1.0 | Ref 1.0 | Ref 1.0 | Ref 1.0 | Ref 1.0 |
| **IMD 2** | 1.00 (0.98 to 1.02) | 1.00 (0.98 to 1.01) | 1,00 (0.98 to 1.02) | 1.00 (0.98 to 1.01) | 0.99(0.98 to 1.01) | 1.00 (0.98 to 1.01) | 0.99 (0.97 to 1.02) | 1.00 (0.98 to 1.02) | 1.00 (0.98,1.02) | 1.00 (0.98,1.01) |
| **IMD 3** | 1.01 (0.99 to 1.03) | 1.02 (1.00 to 1.03)* | 1.02 (1.00 to 1.04)* | 1.03 (1.01 to 1.04)** | 1.02 (1.00 to 1.04) | 1.02 (1.00 to 1.04)* | 1.01 (0.98 to 1.03) | 1.01 (0.99 to 1.03) | 1.02*(1.00,1.04) | 1.02**  (1.01,1.04) |
| **IMD 4** | 0.99 (0.98 to 1.01) | 0.99 (0.98 to 1.01) | 1.02 (1.01 to 1.04)* | 1.03 (1.02 to 1.05)*** | 1.01 (1.00 to 1.03) | 1.01 (0.99 to 1.03)* | 1.01 (0.99 to 1.04) | 1.02 (1.00 to 1.04) | 1.01  (1.00,1.03) | 1.02**  (1.01,1.03) |
| **IMD 5** | 0.95 (0.94 to 0.97)*** | 0.98 (0.96 to 0.99)** | 1.02 (1.00 to 1.04) | 1.05 (1.03 to 1.07)*** | 0.99 (0.98 to 1.01) | 1.02 (1.00 to 1.03)* | 0.96 (0.93 to 0.98)*** | 0.99 (0.96 to 1.01)*** | 1.00  (0.98,1.01) | 1.02**  (1.01,1.04) |
| **White ethnicity** | Ref 1.0 | Ref 1.0 | Ref 1.0 | Ref 1.0 | Ref 1.0 | Ref 1.0 | Ref 1.0 | Ref 1.0 | Ref 1.0 | Ref 1.0 |
| **Black ethnicity** | 0.84 (0.80 to 0.88) *** | 0.82 (0.79 to 0.85)*** | 0.94 (0.90 to 0.98)** | 0.92 (0.88 to 0.95)*** | 0.97 (0.92 to 1.01) | 0.95 (0.92 to 0.99)* | 0.95 (0.89 to 1.03) | 0.92 (0.87 to 0.98)** | 0.97  (0.93,1.01) | 0.96*  (0.92,0.99) |
| **South-Asian ethnicity** | 0.95 (0.92 to 0.98)** | 0.96 (0.94 to 0.99)** | 1.08 (1.05 to 1.12)*** | 1.10 (1.07 to 1.13)*** | 1.11 (1.07 to 1.14)*** | 1.13 (1.10 to 1.16)*** | 1.11 (1.05 to 1.16)** | 1.14 (1.10 to 1.19)*** | 1.11***  (1.08,1.14) | 1.13***  (1.10,1.16) |
| **Mixed/Other ethnicity** |  |  |  |  |  |  |  |  | 0.92***  (0.88,0.96) | 0.95**  (0.91,0.98) |
| **Unknown/Missing ethnicity** |  |  |  |  |  |  |  |  | 0.79***  (0.77,0.80) | 0.74***  (0.73,0.75) |
| **≥ 2 underlying long-term conditions** | 1.54 (1.52 to 1.55)*** | 1.67 (1.66 to 1.69)*** |  |  | 1.37 (1.35 to 1.38)*** | 1.48 (1.47 to 1.50)*** | 1.30 (1.28 to 1.32)*** | 1.42 (1.40 to 1.44)*** | 1.36***  (1.35,1.38) | 1.48***  (1.46,1.49) |
| **≤ 1 underlying long-term condition** | Ref 1.0 | Ref 1.0 |  |  | Ref 1.0 | Ref 1.0 | Ref 1.0 | Ref 1.0 | Ref 1.0 | Ref 1.0 |

# Table S1 Negative binomial regression to compare incidence rate ratios (IRR 95% CI ) for rates of primary care consultations across groups

* p<0.05, ** p<0.01, *** p<0.001

Sensitivity analysis 1 – Cohort of patients with at least two GP consultations with a breathlessness code

Sensitivity analysis 2- Patients with ethnicity coding of "Mixed/Other" and "Unknown/Missing" included

IRR= Incidence rate ratio; IMD = Index of multiple deprivation; MLTC= Multiple long-term conditions; CI= Confidence interval

|  | **Unadjusted IRR** | | **Adjusted IRR (age, sex, ethnicity, IMD)** | | **Adjusted IRR (age, sex, ethnicity, IMD, MLTC)** | | **Sensitivity analysis 1 Adjusted IRR (age, sex, ethnicity, IMD, MLTC)** | | **Sensitivity analysis 2 Adjusted IRR (age, sex, ethnicity, IMD, MLTC)** | |
| --- | --- | --- | --- | --- | --- | --- | --- | --- | --- | --- |
| **Variable** | **Within 6 months** | **Within 24 months** | **Within 6 months** | **Within 24 months** | **Within 6 months** | **Within 24 months** | **Within 6 months** | **Within 24 months** | **Within 6 months** | **Within 24 months** |
| **Age** | 1.01 (1.01 to 1.01)*** | 1.01 (1.01 to 1.01)*** | 1.01 (1.01 to 1.01)*** | 1.01 (1.01 to 1.01)*** | 1.01 (1.01 to 1.01)*** | 1.00 (1.00 to 1.01)*** | 1.01***  (1.00 - 1.01) | 1.00***  (1.00 - 1.00) | 1.01***  (1.01 - 1.01) | 1.00***  (1.00 - 1.01) |
| **Male** | Ref 1.0 | Ref 1.0 | Ref 1.0 | Ref 1.0 | Ref 1.0 | Ref 1.0 | Ref 1.0 | Ref 1.0 | Ref 1.0 | Ref 1.0 |
| **Female** | 0.97 (0.95 to 1.00)* | 1.05 (1.04 to 1.07)*** | 0.99 (0.96 to 1.01) | 1.07 (1.05 to 1.08)*** | 0.98 (0.96 to 1.00) | 1.06 (1.04 to 1.07)*** | 0.98  (0.95 - 1.01) | 1.04***  (1.02 - 1.07) | 0.98*  (0.96 - 1.00) | 1.05***  (1.04 - 1.07) |
| **IMD 1 (lowest deprivation)** | Ref 1.0 | Ref 1.0 | Ref 1.0 | Ref 1.0 | Ref 1.0 | Ref 1.0 | Ref 1.0 | Ref 1.0 | Ref 1.0 | Ref 1.0 |
| **IMD 2** | 0.97 (0.94 to 1.00) | 0.98 (0.96 to 1.00) | 0.97 (0.94 to 1.00) | 0.98 (0.96 to 1.00) | 0.97 (0.94 to 1.00) | 0.98 (0.96 to 1.00) | 0.99  (0.94 - 1.03) | 1.00  (0.96 - 1.03) | 0.97*  (0.94 - 1.00) | 0.98*  (0.96 - 1.00) |
| **IMD 3** | 0.92 (0.89 to 0.95)*** | 0.93 (0.91 to 0.96)*** | 0.93 (0.90 to 0.96)*** | 0.94 (0.92 to 0.96)*** | 0.92 (0.89 to 0.96)*** | 0.94 (0.92 to 0.96)*** | 0.93***  (0.88 - 0.97) | 0.93***  (0.89 - 0.96) | 0.93***  (0.90 - 0.96) | 0.94***  (0.92 - 0.96) |
| **IMD 4** | 0.90 (0.86 to 0.93)*** | 0.92 (0.89 to 0.94)*** | 0.92 (0.89 to 0.95)*** | 0.93 (0.91 to 0.96)*** | 0.91 (0.88 to 0.95)*** | 0.93 (0.90 to 0.95)*** | 0.91***  (0.87 - 0.96) | 0.92***  (0.89 - 0.95) | 0.92***  (0.89 - 0.95) | 0.93***  (0.91 - 0.95) |
| **IMD 5** | 0.92 (0.89 to 0.96)*** | 0.95 (0.92 to 0.97)*** | 0.97 (0.93 to 1.01) | 0.98 (0.96 to 1.01) | 0.96 (0.92 to 0.99)* | 0.97 (0.94 to 0.99)** | 0.94**  (0.90 - 0.99) | 0.96**  (0.93 - 1.00) | 0.96**  (0.92 - 0.99) | 0.97***  (0.94 - 0.99) |
| **White ethnicity** | Ref 1.0 | Ref 1.0 | Ref 1.0 | Ref 1.0 | Ref 1.0 | Ref 1.0 | Ref 1.0 | Ref 1.0 | Ref 1.0 | Ref 1.0 |
| **Black ethnicity** | 0.86 (0.78 to 0.95)** | 0.89 (0.83 to 0.95)** | 0.94 (0.85 to 1.03) | 0.95 (0.88 to 1.01) | 0.96 (0.86 to 1.07) | 0.96 (0.90 to 1.03) | 1.02  (0.88 - 1.19) | 0.97  (0.87 - 1.08) | 0.95  (0.86 - 1.05) | 0.96  (0.90 - 1.03) |
| **South-Asian ethnicity** | 0.98 (0.92 to 1.04) | 1.03 (0.98 to 1.07) | 1.06 (1.00 to 1.13) | 1.09 (1.04 to 1.13)*** | 1.06 (0.99 to 1.13) | 1.10 (1.05 to 1.14)*** | 1.14***  (1.03 - 1.25) | 1.14***  (1.07 - 1.22) | 1.07  (1.00 - 1.13) | 1.10***  (1.05 - 1.15) |
| **Mixed/Other ethnicity** |  |  |  |  |  |  |  |  | 0.97  (0.88 - 1.06) | 1.01  (0.95 - 1.08) |
| **Unknown/Missing ethnicity** |  |  |  |  |  |  |  |  | 0.91***  (0.87 - 0.94) | 0.80***  (0.78 - 0.83) |
| **≥ 2 underlying long-term conditions** | 1.23 (1.20 to 1.26)*** | 1.31 (1.29 to 1.33)*** |  |  | 1.14 (1.12 to 1.17)*** | 1.25 (1.23 to 1.27)*** | 1.12***  (1.08 - 1.16) | 1.24***  (1.21 - 1.28) | 1.14***  (1.11 - 1.16) | 1.24***  (1.22 - 1.26) |
| **≤ 1 underlying long-term condition** | Ref 1.0 | Ref 1.0 |  |  | Ref 1.0 | Ref 1.0 | Ref 1.0 | Ref 1.0 | Ref 1.0 | Ref 1.0 |

# Table S2 Negative binomial regression to compare incidence rate ratios (IRR 95%CI) for rates of routine referrals into secondary care across groups

* p<0.05, ** p<0.01, *** p<0.001

Sensitivity analysis 1 – Cohort of patients with at least two GP consultations with a breathlessness code

Sensitivity analysis 2- Patients with ethnicity coding of "Mixed/Other" and "Unknown/Missing" included

IRR= Incidence rate ratio; IMD = Index of multiple deprivation; MLTC= Multiple long-term conditions; CI= Confidence interval

|  | **Unadjusted IRR** |  | **Adjusted IRR (age, sex, ethnicity, IMD)** | | **Adjusted IRR (age, sex, ethnicity, IMD, MLTC)** | | **Sensitivity analysis 1 Adjusted IRR (age, sex, ethnicity, IMD, MLTC)** | | **Sensitivity analysis 2 Adjusted IRR (age, sex, ethnicity, IMD, MLTC)** | |
| --- | --- | --- | --- | --- | --- | --- | --- | --- | --- | --- |
| **Variable** | **Within 6 months** | **Within 24 months** | **Within 6 months** | **Within 24 months** | **Within 6 months** | **Within 24 months** | **Within 6 months** | **Within 24 months** | **Within 6 months** | **Within 24 months** |
| **Age** | 1.02 (1.02 to 1.02)*** | 1.02 (1.01 to 1.02)*** | 1.02 (1.02 to 1.02)*** | 1.01 (1.01 to 1.02)*** | 1.02 (1.01 to 1.02)*** | 1.01 (1.01 to 1.01)*** | 1.01***  (1.01 - 1.02) | 1.01***  (1.01 - 1.01) | 1.02***  (1.01 - 1.02) | 1.01***  (1.01 - 1.01) |
| **Male** | Ref 1.0 | Ref 1.0 | Ref 1.0 | Ref 1.0 | Ref 1.0 | Ref 1.0 |  |  |  |  |
| **Female** | 0.90 (0.85 to 0.94)*** | 1.01 (0.97 to 1.04) | 0.91 (0.86 to 0.96)*** | 1.03 (0.99 to 1.06) | 0.90 (0.88 to 0.95)*** | 1.02 (0.99 to 1.05) | 0.92**  (0.85 - 0.98) | 1.00  (0.95 - 1.05) | 0.91***  (0.87 - 0.96) | 1.02  (0.99 - 1.05) |
| **IMD 1 (lowest deprivation)** | Ref 1.0 | Ref 1.0 | Ref 1.0 | Ref 1.0 | Ref 1.0 | Ref 1.0 | Ref 1.0 | Ref 1.0 | Ref 1.0 | Ref 1.0 |
| **IMD 2** | 0.96 (0.89 to 1.04) | 0.97 (0.93 to 1.02) | 0.96 (0.90 to 1.04) | 0.97 (0.93 to 1.02) | 0.95 (0.88 to 1.03) | 0.97 (0.93 to 1.02) | 0.87***  (0.78 - 0.97) | 0.94*  (0.87 - 1.00) | 0.97  (0.90 - 1.04) | 0.97  (0.92 - 1.01) |
| **IMD 3** | 0.96 (0.89 to 1.04) | 0.95 (0.90 to 1.00)* | 0.98 (0.91 to 1.05) | 0.96 (0.92 to 1.01) | 0.97 (0.90 to 1.05) | 0.96 (0.91 to 1.01) | 0.94  (0.85 - 1.05) | 0.93*  (0.87 - 1.00) | 0.97  (0.90 - 1.04) | 0.95**  (0.91 - 1.00) |
| **IMD 4** | 0.86 (0.79 to 0.93)*** | 0.89 (0.84 to 0.93)*** | 0.90 (0.83 to 0.97)** | 0.92 (0.87 to 0.97)** | 0.89 (0.82 to 0.96)** | 0.91 (0.86 to 0.96)*** | 0.87**  (0.78 - 0.97) | 0.89***  (0.83 - 0.96) | 0.89***  (0.82 - 0.96) | 0.91***  (0.86 - 0.95) |
| **IMD 5** | 0.84 (0.77 to 0.91)*** | 0.87 (0.83 to 0.92)*** | 0.92 (0.84 to 1.00)* | 0.95 (0.90 to 1.00) | 0.91 (0.83 to 0.98)* | 0.93 (0.88 to 0.98)** | 0.86**  (0.77 - 0.97) | 0.90***  (0.83 - 0.97) | 0.91**  (0.84 - 0.98) | 0.93***  (0.88 - 0.98) |
| **White ethnicity** | Ref 1.0 | Ref 1.0 | Ref 1.0 | Ref 1.0 | Ref 1.0 | Ref 1.0 |  |  |  |  |
| **Black ethnicity** | 0.88 (0.71 to 1.09) | 0.75 (0.64 to 0.87)*** | 1.06 (0.86 to 1.32) | 0.87 (0.75 to 1.01) | 1.07 (0.86 to 1.34) | 0.89 (0.77 to 1.03) | 1.12  (0.81 - 1.55) | 0.89  (0.71 - 1.12) | 1.07  (0.86 - 1.34) | 0.89  (0.77 - 1.03) |
| **South-Asian ethnicity** | 0.97 (0.84 to 1.11) | 0.93 (0.84 to 1.01) | 1.14 (0.99 to 1.31) | 1.07 (0.97 to 1.17) | 1.15 (1.00 to 1.32) | 1.08 (0.99 to 1.18) | 1.38***  (1.13 - 1.68) | 1.24***  (1.09 - 1.42) | 1.15  (1.00 - 1.32) | 1.08  (0.99 - 1.18) |
| **Mixed/Other ethnicity** |  |  |  |  |  |  |  |  | 0.73**  (0.57 - 0.93) | 0.86**  (0.75 - 0.99) |
| **Unknown/Missing ethnicity** |  |  |  |  |  |  |  |  | 0.93  (0.85 - 1.02) | 0.79***  (0.75 - 0.84) |
| **≥ 2 underlying long-term conditions** | 1.34 (1.27 to 1.41)*** | 1.50 (1.45 to 1.56)*** |  |  | 1.16 (1.10 to 1.23)*** | 1.34 (1.29 to 1.39)*** | 1.11***  (1.03 - 1.20) | 1.32***  (1.25 - 1.39) | 1.15***  (1.09 - 1.22) | 1.32***  (1.28 - 1.37) |
| **≤ 1 underlying long-term condition** | Ref 1.0 | Ref 1.0 |  |  | Ref 1.0 | Ref 1.0 | Ref 1.0 | Ref 1.0 | Ref 1.0 | Ref 1.0 |

# Table S3 Negative binomial regression to compare incidence rate ratios (IRR 95%CI) for rates of urgent referrals across groups

* p<0.05, ** p<0.01, *** p<0.001

Sensitivity analysis 1 – Cohort of patients with at least two GP consultations with a breathlessness code

Sensitivity analysis 2- Patients with ethnicity coding of "Mixed/Other" and "Unknown/Missing" included

IRR= Incidence rate ratio; IMD = Index of multiple deprivation; MLTC= Multiple long-term conditions; CI= Confidence interval

|  | **Unadjusted IRR** | | **Adjusted IRR (age, sex, ethnicity, IMD)** | | **Adjusted IRR (age, sex, ethnicity, IMD, MLTC)-** | | **Sensitivity analysis 1 (Adjusted IRR (age, sex, ethnicity, IMD, MLTC)** | | **Sensitivity analysis 2 - Adjusted IRR (age, sex, ethnicity, IMD, MLTC)** | |
| --- | --- | --- | --- | --- | --- | --- | --- | --- | --- | --- |
| **Variable** | **Within 6 months** | **Within 24 months** | **Within 6 months** | **Within 24 months** | **Within 6 months** | **Within 24 months** | **Within 6 months** | **Within 24 months** | **Within 6 months** | **Within 24 months** |
| **Age** | 0.99 (0.99 to 0.99)*** | 1.00 (1.00 to 1.00) *** | 0.99 (0.99 to 0.99)*** | 1.00 (1.00 to 1.00)*** | 0.99 (0.99 to 0.99)*** | 0.99 (0.99 to 1.00)*** | 0.99  (0.99 - 0.99) *** | 1.00  (0.99 - 1.00) *** | 0.99  (0.99 - 0.99) *** | 0.99  (0.99 - 1.00) *** |
| **Male** | Ref 1.0 | Ref 1.0 | Ref 1.0 | Ref 1.0 | Ref 1.0 | Ref 1.0 |  |  |  |  |
| **Female** | 1.10 (1.05 to 1.15)*** | 1.03 (1.00 to 1.06)* | 1.05 (1.00 to 1.10) | 1.01 (0.98 to 1.04) | 1.04 (1.00 to 1.10) | 1.01 (0.98 to 1.04) | 0.99  (0.93 - 1.05) | 0.96  (0.92 - 1.00) ** | 1.06  (1.01 - 1.10) ** | 1.02  (0.99 - 1.05) |
| **IMD 1 (lowest deprivation)** | Ref 1.0 | Ref 1.0 | Ref 1.0 | Ref 1.0 | Ref 1.0 | Ref 1.0 | Ref 1.0 | Ref 1.0 | Ref 1.0 | Ref 1.0 |
| **IMD 2** | 0.99 (0.92 to 1.05) | 0.99 (0.95 to 1.04) | 0.98 (0.91 to 1.05) | 0.99 (0.95 to 1.04) | 0.98 (0.91 to 1.05) | 0.99 (0.94 to 1.03) | 0.97  (0.88 - 1.07) | 1.00  (0.93 - 1.07) | 0.98  (0.92 - 1.04) | 0.98  (0.94 - 1.03) |
| **IMD 3** | 0.99 (0.93 to 1.07) | 1.06 (1.02 to 1.11)** | 0.97 (0.91 to 1.04) | 1.05 (1.01 to 1.10)* | 0.97 (0.91 to 1.04) | 1.05 (1.00 to 1.10)* | 1.00  (0.90 - 1.10) | 1.10  (1.03 - 1.18) *** | 0.97  (0.90 - 1.03) | 1.04  (1.00 - 1.09) * |
| **IMD 4** | 0.95 (0.88 to 1.02) | 1.06 (1.01 to 1.11)* | 0.91 (0.84 to 0.97)*** | 1.03 (0.99 to 1.08) | 0.91 (0.84 to 0.97)** | 1.03 (0.98 to 1.07) | 0.90  (0.81 - 1.00) ** | 1.06  (0.99 - 1.13) | 0.90  (0.84 - 0.96) *** | 1.02  (0.97 - 1.06) |
| **IMD 5** | 1.03 (0.96 to 1.11) | 1.15 ( 1.10 to 1.21)*** | 0.95 (0.88 to 1.02) | 1.11 (1.06 to 1.16)*** | 0.95 (0.89 to 1.02) | 1.09 (1.04 to 1.14)** | 0.93  (0.84 - 1.03) | 1.09**  (1.02 - 1.17) | 0.94  (0.88 - 1.01) | 1.08  (1.03 - 1.13) *** |
| **White ethnicity** | Ref 1.0 | Ref 1.0 | Ref 1.0 | Ref 1.0 | Ref 1.0 | Ref 1.0 | Ref 1.0 | Ref 1.0 | Ref 1.0 | Ref 1.0 |
| **Black ethnicity** | 1.53 (1.29 to 1.80)*** | 1.39 (1.24 to 1.56)*** | 1.42 (1.20 to 1.67)*** | 1.31 (1.17 to 1.47)*** | 1.43 (1.21 to 1.69)*** | 1.33 (1.19 to 1.50)*** | 1.55  (1.20 - 2.01) *** | 1.36  (1.13 - 1.63) *** | 1.42  (1.20 - 1.68) *** | 1.33  (1.19 - 1.49) *** |
| **South-Asian ethnicity** | 1.55 (1.39 to 1.73)*** | 1.38 ( 1.28 to 1.49)*** | 1.41 (1.26 to 1.57)*** | 1.32 (1.23 to 1.43)*** | 1.42 (1.27 to 1.58)*** | 1.34 (1.25 to 1.45)*** | 1.40  (1.17 - 1.67)*** | 1.46  (1.30 - 1.65) *** | 1.41  (1.26 - 1.57) *** | 1.34  (1.24 - 1.44) *** |
| **Mixed/Other ethnicity** |  |  |  |  |  |  |  |  | 1.11  (0.93 - 1.31) | 1.07  (0.95 - 1.20) |
| **Unknown/Missing ethnicity** |  |  |  |  |  |  |  |  | 1.07  (0.94 - 1.21) | 0.82  (0.75 - 0.89) *** |
| **≥ 2 underlying long-term conditions** | 1.00 (0.96 to 1.05) | 1.21 (1.18 to 1.26)*** |  |  | 1.14 (1.08 to 1.20)*** | 1.31 (1.27 to 1.35)*** | 1.16  (1.08 - 1.25) *** | 1.32  (1.25 - 1.38) *** | 1.13  (1.08 - 1.19) *** | 1.30  (1.26 - 1.34) *** |
| **≤ 1 underlying long-term condition** | Ref 1.0 | Ref 1.0 |  |  | Ref 1.0 | Ref 1.0 | Ref 1.0 | Ref 1.0 | Ref 1.0 | Ref 1.0 |

# Table S4 Negative binomial regression to compare Incidence rate ratios (IRR 95%CI) for rates of hospital admissions across groups

* p<0.05, ** p<0.01, *** p<0.001

Sensitivity analysis 1 – Cohort of patients with at least two GP consultations with a breathlessness code

Sensitivity analysis 2- Patients with ethnicity coding of "Mixed/Other" and "Unknown/Missing" included IRR= Incidence rate ratio; IMD = Index of multiple deprivation; MLTC= Multiple long-term conditions

IRR= Incidence rate ratio; IMD = Index of multiple deprivation; MLTC= Multiple long-term conditions; CI= Confidence interval

|  | **Unadjusted OR** | | **Adjusted OR (age, sex, ethnicity, IMD)** | | **Adjusted OR (age, sex, ethnicity, IMD, MLTC)** | | **Adjusted RR (age, sex, ethnicity, IMD, MLTC)** | | **Sensitivity analysis 1 Adjusted OR (age, sex, ethnicity, IMD, MLTC)** | | **Sensitivity analysis 2 Adjusted OR (age, sex, ethnicity, IMD, MLTC)** | |
| --- | --- | --- | --- | --- | --- | --- | --- | --- | --- | --- | --- | --- |
| **Variable** | **Within 6 months** | **Within 24 months** | **Within 6 months** | **Within 24 months** | **Within 6 months** | **Within 24 months** | **Within 6 months** | **Within 24 months** | **Within 6 months** | **Within 24 months** | **Within 6 months** | **Within 24 months** |
| **Age** | 1.02 (1.02 to 1.012)*** | 1.02 (1.02 to 1.02)*** | 1.02 (1.02 to 1.02)*** | 1.02 (1.02 to 1.02)*** | 1.02 (1.02 to 1.02)*** | 1.02 (1.01 to 1.02)*** | 1.01***  (1.01 - 1.01) | 1.01***  (1.01 - 1.01) | 1.01  (1.01 - 1.01) *** | 1.01  (1.01 - 1.01) *** | 1.02  (1.02 - 1.02) *** | 1.02  (1.01 - 1.02) *** |
| **Male** | Ref 1.0 | Ref 1.0 | Ref 1.0 | Ref 1.0 | Ref 1.0 | Ref 1.0 |  |  |  |  |  |  |
| **Female** | 0.81 (0.79 to 0.84)*** | 0.91 (0.89 to 0.94)*** | 0.82 (0.80 to 0.84)*** | 0.91 (0.89 to 0.94)*** | 0.81 (0.79 to 0.84)*** | 0.90 (0.87 to 0.92)*** | 0.87***  (0.85 - 0.88) | 0.94***  (0.93 - 0.96) | 0.78  (0.75 - 0.81) *** | 0.82  (0.79 - 0.85) *** | 0.82  (0.80 - 0.84) *** | 0.90  (0.88 - 0.93) *** |
| **IMD 1 (lowest deprivation)** | Ref 1.0 | Ref 1.0 | Ref 1.0 | Ref 1.0 | Ref 1.0 | Ref 1.0 | Ref 1.0 | Ref 1.0 | Ref 1.0 | Ref 1.0 | Ref 1.0 | Ref 1.0 |
| **IMD 2** | 1.07 (1.02 to 1.11)** | 1.07 (1.03 to 1.12)** | 1.07 (1.02 to 1.12)** | 1.08 (1.03 to 1.12)*** | 1.07 (1.02 to 1.12)** | 1.08 (1.03 to 1.12)*** | 1.05  (1.02 - 1.08)*** | 1.04  (1.02 - 1.07)*** | 1.08  (1.01 - 1.15**) | 1.11  (1.04 - 1.18) *** | 1.07  (1.03 - 1.12) *** | 1.08  (1.04 - 1.12) *** |
| **IMD 3** | 1.08 (1.03 to 1.13)** | 1.10 (1.06 to 1.14)*** | 1.11 (1.06 to 1.16)*** | 1.13 (1.08 to 1.18)*** | 1.11 (1.06 to 1.16)*** | 1.12 (1.08 to 1.17)*** | 1.07  (1.04 - 1.11)*** | 1.07  (1.04 - 1.09)*** | 1.13  (1.06 - 1.21) *** | 1.18 (1.10 - 1.25) *** | 1.11  (1.07 - 1.16) *** | 1.12  (1.08 - 1.17) *** |
| **IMD 4** | 1.15 (1.10 to 1.20)*** | 1.20 (1.15 to 1.25)*** | 1.22 (1.16 to 1.27)*** | 1.27 (1.22 to 1.33)*** | 1.21 (1.15 to 1.27)*** | 1.26 (1.20 to 1.31)*** | 1.14  (1.11 - 1.18)*** | 1.13  (1.11 - 1.16)*** | 1.30  (1.21 - 1.38) *** | 1.38  (1.30 - 1.48) *** | 1.22  (1.16 - 1.27) *** | 1.26  (1.21 - 1.31) *** |
| **IMD 5** | 1.26 (1.20 to 1.32)*** | 1.29 (1.24 to 1.35)*** | 1.41 (1.35 to 1.48)*** | 1.46 (1.40 to 1.53)*** | 1.40 (1.33 to 1.47)*** | 1.42 (1.36 to 1.49)*** | 1.26 (1.22 - 1.30)*** | 1.21 (1.18 - 1.24)*** | 1.48  (1.38 - 1.58) *** | 1.55  (1.45 - 1.66) *** | 1.40  (1.34 - 1.47) *** | 1.42  (1.36 - 1.48) *** |
| **White ethnicity** | Ref 1.0 | Ref 1.0 | Ref 1.0 | Ref 1.0 | Ref 1.0 | Ref 1.0 | Ref 1.0 | Ref 1.0 | Ref 1.0 | Ref 1.0 | Ref 1.0 | Ref 1.0 |
| **Black ethnicity** | 0.66 (0.59 to 0.75)*** | 0.70 (0.63 to 0.78)*** | 0.72 (0.64 to 0.82)*** | 0.75 (0.68 to 0.84)*** | 0.73 (0.65 to 0.83)*** | 0.78 (0.70 to 0.87)*** | 0.79 (0.72 - 0.88)*** | 0.86 (0.81 - 0.92)*** | 0.65  (0.54 - 0.79) *** | 0.68  (0.56 - 0.81) *** | 0.73  (0.65 - 0.83) *** | 0.78***  (0.70 - 0.87) |
| **South-Asian ethnicity** | 0.65 (0.60 to 0.71)*** | 0.70 (0.65 to 0.75)*** | 0.75 (0.69 to 0.82)*** | 0.81 (0.75 to 0.87)*** | 0.76 (0.69 to 0.82)*** | 0.82 (0.77 to 0.89)*** | 0.81 (0.76 - 0.87)*** | 0.89 (0.85 - 0.93)*** | 0.65  (0.57 - 0.74) *** | 0.69  (0.61 - 0.78) *** | 0.76  (0.69 - 0.82)*** | 0.82  (0.76 - 0.89) *** |
| **Mixed/Other ethnicity** |  |  |  |  |  |  |  |  |  |  | 0.74  (0.66 - 0.84) *** | 0.75  (0.67 - 0.83) *** |
| **Unknown/Missing ethnicity** |  |  |  |  |  |  |  |  |  |  | 0.78  (0.75 - 0.82) *** | 0.67  (0.64 - 0.70) *** |
| **≥ 2 underlying long-term conditions** | 1.37 (1.33 to 1.42)*** | 1.68 (1.64 to 1.73)*** |  |  | 1.15 (1.12 to 1.19)*** | 1.44 (1.39 to 1.48)*** | 1.11 (1.08 -1.13)*** | 1.23 (1.21 - 1.25)*** | 0.99  (0.95 - 1.04) | 1.21  (1.16 - 1.27) *** | 1.14  (1.10 - 1.17) *** | 1.41  (1.37 - 1.45) *** |
| **≤ 1 underlying long-term condition** | Ref 1.0 | Ref 1.0 |  |  | Ref 1.0 | Ref 1.0 | Ref 1.0 | Ref 1.0 | Ref 1.0 | Ref 1.0 | Ref 1.0 | Ref 1.0 |

# Table S5 Logistic regression to compare likelihood of diagnosis (OR 95%CI) across groups

* p<0.05, ** p<0.01, *** p<0.001

Sensitivity analysis 1 – Cohort of patients with at least two GP consultations with a breathlessness code

Sensitivity analysis 2- Patients with ethnicity coding of "Mixed/Other" and "Unknown/Missing" included

OR= Odds ratio; RR= Relative risk ratio; IMD = Index of multiple deprivation; MLTC= Multiple long-term conditions; CI= Confidence interval
